# EXPERIENCES OF RACISM IN HEALTH AND LIFE-COURSE RESEARCH: A MEASUREMENT BOOKLET AND RECOMMENDATIONS

**DOI:** 10.1093/geroni/igad104.3325

**Published:** 2023-12-21

**Authors:** James Iveniuk, Monica Peek

**Affiliations:** NORC at the University of Chicago, Chicago, Illinois, United States; University of Chicago, Chicago, Illinois, United States

## Abstract

Numerous, interconnected fields of research study racism, and health inequities emerging from systemic racism. Researchers who are interested in strengthening this work may, however, find themselves at a loss as to where to begin. They may not have a conceptual framework that informs their choice of measures and approaches. They may also lack awareness of appropriate measures, that they can choose from to create impactful work. To meet this need, we create a research-community asset for the measurement of racial identity, intrapersonal/internalized racism, interpersonal racism, institutional racism, and structural racism, with an emphasis on measuring these experiences and their impact on health across the life course. Where applicable, we provide guidance on scoring. The asset is prescriptive in what measures it offers to readers, and in terms of the multi-level theoretical framework that we use to inform our selection. However it is also agnostic about health outcomes or research designs, and we provide measures that are applicable to self-report and administrative data sources, in order to be applicable to as many different researchers as possible, both in academic and community-engaged settings. We also provide some general guidance for translation into different languages, to be inclusive of respondents from diverse linguistic backgrounds.

